# Personalized Medicine Through Semisolid-Extrusion Based 3D Printing: Dual-Drug Loaded Gummies for Enhanced Patient Compliance

**DOI:** 10.1007/s11095-024-03813-z

**Published:** 2025-01-17

**Authors:** Aditi Holkunde, Indrajeet Karnik, Prateek Uttreja, Nagarjuna Narala, Honghe Wang, Rasha M. Elkanayati, Sateesh Kumar Vemula, Michael A. Repka

**Affiliations:** 1 Department of Pharmaceutics and Drug Delivery, School of Pharmacy, The University of Mississippi, University, MS 38677 USA; 2 Department of Pharmaceutics, School of Pharmaceutical Sciences, Lovely Professional University, Phagwara, Punjab 144411 India; 3 Pii Center for Pharmaceutical Technology, The University of Mississippi, University, MS 38677 USA

**Keywords:** 3D printing, Drug-loaded gummies, Isoniazid, Personalized medicine, Pyridoxine, Semisolid extrusion, Tuberculosis treatment

## Abstract

**Purpose:**

The purpose of this research was to develop and characterize dual-drug Isoniazid-Pyridoxine gummies using Semisolid Extrusion (SSE) 3D printing technology, aimed at personalized dosing for a broad patient demographic, from pediatric to geriatric. This study leverages SSE 3D printing, an innovative approach in personalized medicine, to enable precise dose customization and improve patient adherence. By formulating dual drug-loaded gummies, the research addresses the challenges of pill burden and poor palatability associated with traditional tuberculosis regimens, ultimately enhancing the therapeutic experience and effectiveness for patients across various age groups.

**Methods:**

Gummies were formulated using varying ratios of gelatin, carrageenan, and xylitol, and printed using the BIO X 3D printer. Rheological properties were evaluated to confirm printability, shear-thinning behavior, and viscosity recovery. *In vitro* drug release and stability were assessed under refrigerated (5 ± 3°C) and ambient (25 ± 2°C) storage conditions. FT-IR spectroscopy was used to examine drug-excipient interactions.

**Results:**

The optimized F3 formulation, containing 900 mg Isoniazid and 30 mg Pyridoxine, demonstrated successful printability and structural integrity. Over 80% of both drugs were released within 30 min. Rheological testing confirmed ideal shear-thinning and viscoelastic properties for extrusion-based printing. Suitable textural properties for pediatric patient compliance were observed. Stability studies showed that both drug content and release profiles remained consistent for 30 days under refrigerated storage.

**Conclusions:**

This study determines the potential of SSE 3D printing in fabricating personalized Isoniazid-Pyridoxine-loaded gummies, offering a novel, patient-friendly dosage form for tuberculosis treatment. The optimized formulation exhibited excellent printability, stability, and rapid drug release, positioning 3D-printed gummies as a promising alternative to conventional oral dosage forms in enhancing patient adherence.

**Graphical abstract:**

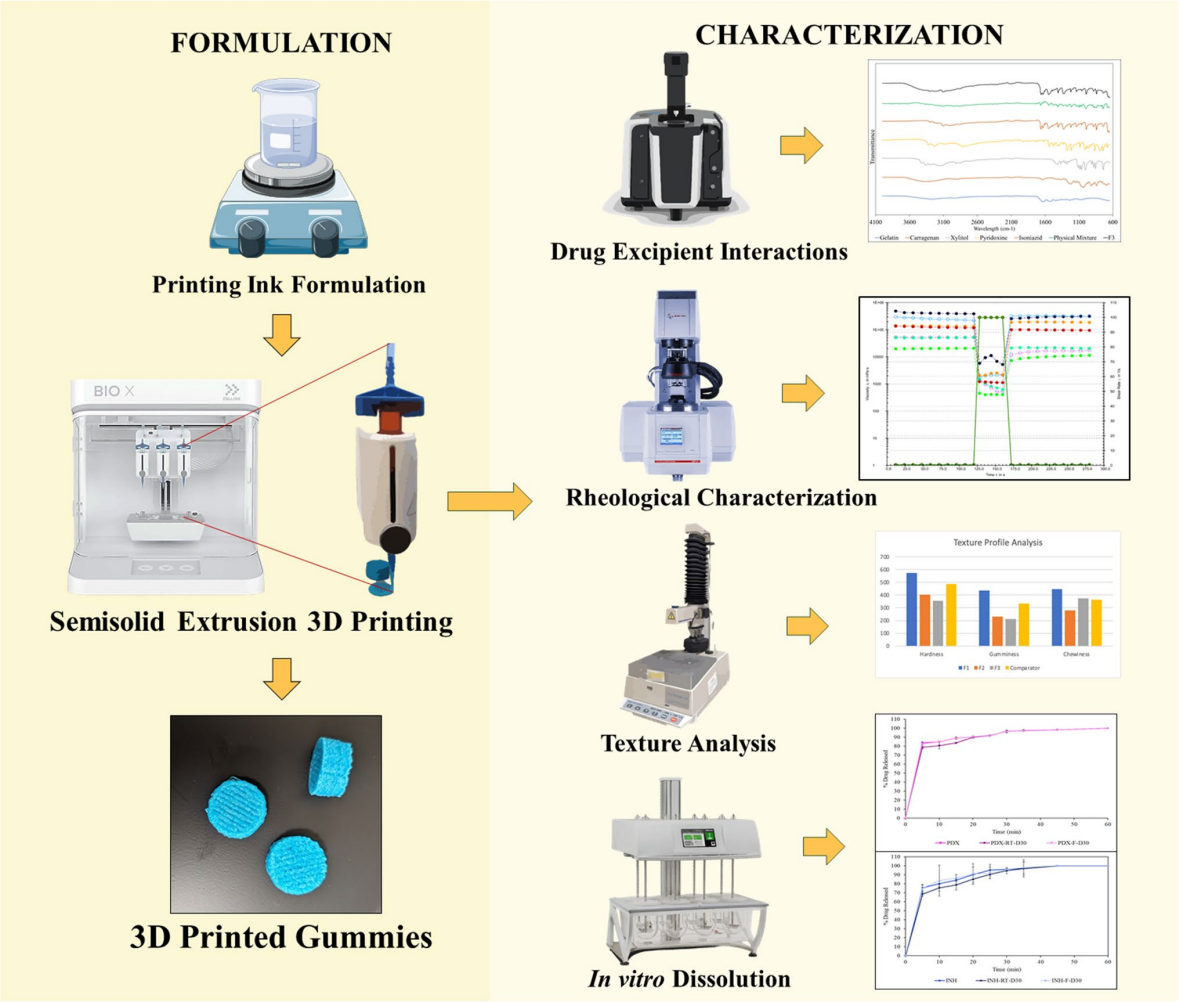

## Introduction

Tuberculosis (TB), caused by *Mycobacterium tuberculosis*, is a major public health issue, responsible for over 10 million new cases and 1.5 million deaths annually, making it the leading cause of death from a single infectious agent [1, 2]. While TB primarily affects the lungs, it can spread to other organs and is transmitted via airborne droplets from coughing or sneezing. With an estimated 1.7 billion people carrying latent TB infection, preventing its progression to active disease is a major global health priority [3]. The World Health Organization (WHO) emphasizes the importance of Tuberculosis Preventive Treatment (TPT), particularly for high-risk populations, including individuals with HIV and close contacts of active TB patients [4]. Isoniazid (INH) remains a cornerstone of TPT, but the lack of child-friendly formulations and difficulties in administering the drug, particularly in pediatric and geriatric populations, present significant barriers to effective treatment [5–7].

Pediatric and geriatric patients often face unique challenges with existing TB treatments [8]. Liquid formulations of INH, though available, require precise measurement, increasing the risk of dosing errors, and often contain preservatives that may cause adverse effects, such as dental issues in children [9]. Moreover, the 10-mg/mL INH syrup is helpful for younger children but older children who have trouble swallowing tablets would need to use enormous amounts to get the recommended daily dosage due to the syrup's low concentration [10]. Crushing INH tablets to combine with fluids is a common practice in the absence of liquid formulations, but this compromises the stability and taste of the medication, leading to poor palatability and inconsistent dosing. ​The instability of the crushed tablets, along with possible uneven distribution in suspensions, exacerbates dosing inconsistencies, increasing the risk of noncompliance and the emergence of drug resistance [11].

INH therapy also presents the risk of peripheral neuropathy, and neurotoxicity caused by various anti-tubercular drugs. Additionally, the likelihood of peripheral neuropathy is nearly four times higher in individuals with drug-resistant TB, HIV infection, malnutrition, diabetes mellitus (DM), alcoholism, chronic renal failure, or advanced age [12]. Peripheral neuropathy can be prevented in these susceptible individuals through the simultaneous administration of pyridoxine (PDX) supplements [13]. WHO guidelines advocate for the inclusion of PDX in TB regimens containing INH, particularly for patients at risk of developing neurological complications [14]. However, the need to administer multiple drugs for a single condition adds to the pill burden, further reducing adherence in populations that already struggle with complex treatment regimens [15, 16]. Non-adherence is a significant barrier to effective TB treatment, with reasons ranging from the complexity and length of therapy to adverse drug effects and poor palatability of formulations [16]. These issues underscore the urgent need for age-appropriate, patient-friendly drug formulations that can simplify regimens and enhance compliance.

Innovative drug delivery technologies, such as 3D-printed chewable dosage forms, offer a promising solution to these challenges. Chewable gummies can mask the bitter taste of medications, eliminate the need for water during administration, and can be easily customized to provide precise dosing for different patient groups [17]. Recent advancements in 3D printing technology, particularly semisolid extrusion (SSE), have revolutionized the ability to produce personalized drug formulations [17–19]. This method allows for the creation of customized dosage forms with precise drug release profiles, improving both patient acceptability and adherence [20]. Furthermore, 3D-printed medications have been shown to improve patient perceptions, making treatment more acceptable and enhancing compliance [21]. Combining INH and PDX into a single, chewable gummy has the potential to reduce pill burden, simplify TB treatment regimens, and improve therapeutic outcomes in pediatric and geriatric populations, where adherence is often a critical issue.

Semisolid extrusion (SSE) 3D printing is a promising technology for personalized medicine, enabling tailored formulations to meet individual patient needs and precise dosage adjustments [22, 23]. This material extrusion technique deposits gel or paste in layers to create 3D objects, with the material hardening to support subsequent tiers. When contrasted with alternative extrusion-based methods like Fused Deposition Modelling or Direct Powder Extrusion, SSE 3D printing distinguishes itself through its utilization of low printing temperatures, rendering it particularly well-suited for applications in drug delivery and biomedicine. Additionally, the use of disposable syringes, which hold semi-solid feedstock or printing inks, brings advantages in ensuring compliance with stringent quality standards essential for pharmaceutical applications [22]. Gummy-like printlets prepared by SSE have been demonstrated to have greater appeal among children than printlets prepared using other technologies [24]. SSE 3D Printing has been used in a clinical setting to manufacture personalized medicine to control isoleucine levels in children with maple syrup urine disease [6]. The low printing temperatures, flexible design features, and patient compliance make SSE particularly well-suited for creating personalized, patient-friendly formulations such as gummies, which offer precise dosage control and improved patient compliance.

The study aims to formulate and evaluate Isoniazid-Pyridoxine-loaded gummies for use in a population ranging from pediatric to geriatric and to study the applicability of BIO X 3D printer in printing gummy formulations.

## Materials and Methods

### Materials

Isoniazid (INH) and Pyridoxine Hydrochloride (PDX) were purchased from Cayman Chemical (Ann Arbor, MI, USA). Gelatin from porcine skin gel strength 300, Type was purchased from Sigma-Aldrich GmbH. Carrageenan was gifted by AEP Colloids. Xanthan Gum (XANATURAL 180) and Pectin type D slow set-Z were purchased from CP Kelco. Benecel™ K15M (HPMC) was gifted by Ashland (Wilmington, DE, USA). Butylated Hydroxy Anisole (BHA) and Butylated Hydroxytoluene (BHT) were purchased from MP Biomedicals, LLC. The food colors red, and blue were purchased from PCCA. Xylitol powder (Xylisorb 300) was purchased from Roquette America, Inc. Pepsin from porcine gastric mucosa, powder, 250 units/mg was purchased from Sigma-Aldrich GMBH. The reagents and solvents used were of analytical grade and were purchased from Fischer Scientific (Fair Lawn, NJ, USA). Scintillation vials and centrifuge tubes were purchased from Fischer Scientific (Hampton, NH, USA).

### Methods

#### Preparation of Gummy Matrix

To assess the printability of selected polymers, the placebos were prepared and printed before making the formulations. The placebos were prepared using two different methods; the first method involves the mixing of all ingredients directly into the gelatin solution (Component A). In contrast, the second method involves the preparation of two separate components (A and B), with the carrageenan paste forming component B, which is then combined with component A. The methods are elaborated in Table I.

**Table I:** Methods of Preparation of Gummy Matrix

Method	Direct addition (Method 1)	Carrageenan Paste Method (Method 2)
Step 1	Weighed gelatin added to water and hydrated for 20 min at room temperature. The mixture was heated at 80°C for 30 min until gelatin dissolved. (Component A)	
Step 2	Carrageenan, INH, PDX, and Xylitol are directly added to the gelatin solution and stirred for 30 min at 80°C	Carrageenan is stirred in water to form a paste (Component B), then added to the gelatin solution. INH, PDX, and Xylitol are added afterward and stirred for 30 min at 80°C
Step 3	The final mixture is transferred to 9 pneumatic syringes (3 mL capacity) and kept at room temperature for 24 h for polymer hydration	

#### Screening for Printability

The prepared samples/ placebos were tested for printability with a broad range of formulation components as shown in Table II. Gelatin and pectin were used as gelling agents. HPMC, Xanthan gum, and carrageenan were used as viscosity modifiers. Xylitol is used as a sweetening agent. Placebos were prepared using the above-listed method and were tested for printability. Based on preliminary process evaluation, printing was conducted at three different temperature–pressure combinations: 30°C with 200 kPa, 35°C with 250 kPa, and 40°C with 300 kPa.

**Table II:** Composition of Placebos

	Purified water (gm)	Gelatin (gm)	Pectin (gm)	HPMC (gm)	Xanthan gum (gm)	Carrageenan (gm)	Carrageenan (gm) + Purified water (mL)	BHA-BHT (gm)	Xylitol (gm)
P1	8	1.9	-	-	-	0.1	-	0.14–0.014	4.2
P2	8	1.8	-	-	-	0.1	-	0.14–0.014	4.2
P3	8	1.6	-	-	-	0.1	-	0.14–0.014	4.2
P4	8	1.5	-	-	-	0.1	-	0.14–0.014	4.2
P5	8	-	1.9	-	-	0.1	-	0.14–0.014	4.2
P6	8	-	1.8	-	-	0.1	-	0.14–0.014	4.2
P7	8	-	1.6	-	-	0.1	-	0.14–0.014	4.2
P8	8	-	1.5	-	-	0.1	-	0.14–0.014	4.2
P9	12	1.5	-	0.1	-	-	-	0.14–0.014	0.2
P10	10	1.5	-	0.1	-	-	-	0.14–0.014	2.2
P11	8	1.5	-	0.1	-	-	-	0.14–0.014	4.2
P12	6	1.5	-	0.1	-	-	-	0.14–0.014	6.2
P13	12	1.5	-	-	0.1	-	-	0.14–0.014	0.2
P14	10	1.5	-	-	0.1	-	-	0.14–0.014	2.2
P15	8	1.5	-	-	0.1	-	-	0.14–0.014	4.2
P16	6	1.5	-	-	0.1	-	-	0.14–0.014	6.2
P17	12	1.5	-	-	-	0.1	-	0.14–0.014	0.2
P18	10	1.5	-	-	-	0.1	-	0.14–0.014	2.2
P19	8	1.5	-	-	-	0.1	-	0.14–0.014	4.2
P20	6	1.5	-	-	-	0.1	-	0.14–0.014	6.2
P21	10	1.5	-	-	-	-	0.1 + 3	0.14–0.014	2.2
P22	8	1.6	-	-	-	-	0.1 + 3	0.14–0.014	4.2

#### 3D-printing Process and Parameters

Gummies were manufactured using a BIO X 3D Printer (CELLINK AB, Gothenburg, SE 41346, Sweden). The gummies were printed 24 h after the gelatin matrix preparation to ensure the polymers' hydration. The different shapes of gummies were designed using Autodesk Tinkercad software (Tinkercad, Autodesk Inc, CA, USA) and exported as a stereolithography file (.stl). Following the findings from the placebo studies, the printing parameters outlined in Table III were chosen for 3D printing formulations with 300 mg of INX and 10 mg of PDX per unit dose. Table IV shows the target dimensions of the cylindrical model. The formulations F1-F3 were designed to get the gummies' target weight and formulations F3A-F3D were designed to study the effect of varying the ratio of gelatin and carrageenan in the formulations (Table IV).

**Table III:** Printing Parameters for Formulations

Printing Parameters	F1	F2	F3
Printing Pressure (kPa)	200–130	200–160	200–180
Nozzle Diameter (mm)	0.4		
Printing temperature (°C)	30		
Infill density (%)	25		
Printing speed (mm/s)	10.0		
Layer Height (mm)	0.4

**Table IV:** Composition and Target Dimensions of 3D-Printed Gummies

Formulation	INH (mg)	PDX (mg)	Purified water (gm)	Gelatin (gm)	Carrageenan (gm) + Purified water (mL)	BHA-BHT (gm)	Xylitol (gm)	Units printed from a single cartridge	Target Weight of Gummy (gm)	Dimensions of Designed Gummy (mm)
F1	300	10	10	1.5	0.1 + 3	0.14–0.014	4.2	1	3.6	16 × 16x9
F2	600	20	10	1.5	0.1 + 3	0.14–0.014	4.2	2	1.8	15 × 15x8
F3	900	30	10	1.5	0.1 + 3	0.14–0.014	4.2	3	1.2	14 × 14x7
F3A	900	30	10	1.43	0.16 + 3	0.14–0.014	4.2	3	1.2	14 × 14x7
F3B	900	30	10	1.38	0.20 + 3	0.14–0.014	4.2	3	1.2	14 × 14x7
F3C	900	30	10	1.35	0.24 + 3	0.14–0.014	4.2	3	1.2	14 × 14x7
F3D	900	30	10	1.32	0.27 + 3	0.14–0.014	4.2	3	1.2	14 × 14x7

### Evaluation of Gummies

The gummies were evaluated for their physical appearance, weight uniformity, drug content, *in vitro* release, texture profile, rheology of the gummy matrix, and stability.

#### Appearance, Dimensions, and Weight

The physical appearance of all the printed gummies was investigated. The height, length, and width were measured using a digital caliper (Fisher brand™ Traceable™ Digital Calipers). Printed gummies were weighed individually on an analytical balance (Mettler Toledo® -ME54TE—Analytical Balance). All measurements were carried out in triplicates.

#### Rheological Tests for Gummy Matrix

The rheological analysis of gummy matrix samples was conducted using a Modular Compact Rheometer (MCR) 120 (Anton Paar, Graz, Austria), with a cone plate (25 mm diameter, 1 mm gap) and a controlled convection/radiant heating oven for precise temperature regulation. The formulations were immersed in a 30°C bath for 10 min before testing to achieve a more fluid state, facilitating handling without significantly compromising the internal structure and minimizing air entrapment.

Shear-viscosity tests were performed in flow ramp mode, with the shear rate escalating from 0.01 to 100 s⁻^1^ over 120 s at 30°C. Thixotropy was assessed at 30°C via shear recovery tests comprising three distinct phases: an initial low shear rate of 0.4 s⁻^1^ for 120 s, succeeded by a high shear rate of 100 s⁻^1^ for 40 s, and concluding with a low shear rate of 0.4 s⁻^1^ for 120 s. The structural regeneration of inks was quantified as the percentage of viscosity measured during the initial 40 s and the 120 s in the third phase (after a high shear rate), relative to the average viscosity recorded in the final 40 s of the first stage, where equilibrium viscosity was attained.

Frequency sweep investigations were conducted at 30°C, with angular frequency varying from 0.1 to 10 rad/s at a constant deformation of 0.5% strain (within the linear viscoelastic range, LVR). In each test, the mean data from three replicates were utilized to construct the curves. The RheoCompass program (Anton Paar Software, Graz, Austria) was used to record and process the results [25].

#### Fourier Transform Infrared (FT-IR) Spectroscopy

The assessment of the compatibility between drugs and excipients in the mixtures and formulations was conducted utilizing a Cary-630 FTIR spectrometer (Agilent Technologies, Santa Clara, CA, USA). Sampling was performed utilizing the MIRacle Attenuated Total Reflection system (Pike Technologies MIRacle ATR, Madison, WI, USA), which features a single-bounce, diamond-coated Zinc Selenide internal reflection element. The infrared spectra of the samples were gathered within the wavenumber range of 650–4000 cm^−1^ [26].

#### Drug Content

Three gummies, each weighing approximately 1.2 g, were dissolved in 500 ml of Milli-Q water in a volumetric flask. The combination underwent sonication using a Bransonic® ultrasonic cleaner from the USA for 40 min. Ultimately, the supernatant underwent filtration via a nylon membrane filter (0.45 μm) and was subsequently diluted with 0.1 N hydrochloric acid and water (3 in 100) for INH [27], while dilute hydrochloric acid (1 in 100) was used for PDX [28]. The amount of each drug was measured at 216 nm (INH) and 258 nm (PDX) using a UV–Vis spectrophotometer (GENESYS 120 version 2.5).

#### Texture Analysis

A Texture Analyzer TA.XT2i (Texture Technologies Corp. and Stable Micro Systems, Ltd. Hamilton, MA) equipped with a 7 mm diameter, 45 mm long stainless -steel Probe (TA-57) was used to determine the texture properties of the printed gummies. To simulate the chewing process, samples were placed below the probe and compressed in two successive cycles at a speed of 1 mm/sec. The pre-test and post-test speeds were set to 1.5 mm/sec, with a trigger force of 5 g (A). A 5-s interval was allowed between the first and second compressions, and samples were compressed to a distance of 5 mm. Each sample was tested in triplicate, and textural properties such as hardness, springiness, and chewiness were compared against a commercial comparator product. Data analysis was performed using Exponent software (version 6.1.5.0) (*n* = 5).

#### *In Vitro* Drug Release

*In vitro* drug release studies were conducted for the 3D-printed gummies to evaluate the drug release profiles from the formulations utilizing a Type II dissolution apparatus (ERWEKA light, ERWEKA GmBH). Samples weighing between 1.18—1.20 g were immersed in vessels filled with 900 mL of 0.1 N hydrochloric acid (pH 1.2) at a temperature of 37 ± 0.5°C, with paddles rotating at 100 rpm. According to the guidelines provided by USP, a specific amount of pepsin should be used to achieve an activity not exceeding 750,000 units/L in the dissolution medium to effectively digest the gelatin present in the formulation [29]. Spiral sinkers were used to ensure the gummies remained submerged during testing. Samples of the release media were collected using a syringe, subsequently filtered, and analyzed at 5, 10, 15, 20, 25, 30, 35, 45, and 60-min intervals. The samples obtained were subjected to dilution and subsequently analyzed for their concentration utilizing a UV–Vis spectrophotometer (GENESYS 120 version 2.5) at wavelengths of 216 nm for PDX and 258 nm for INH. Every formulation underwent analysis in triplicate.

#### Stability Studies

The gummies were stored at 25 ± 2°C and refrigerated temperature (5 ± 3°C) for a 30-day stability study. The 3D-printed gummies were evaluated for their physicochemical characteristics at scheduled time intervals i.e. day 0 and day 30 for the cartridges and day 1, day 3, and day 30 for the 3D-printed gummy formulations. Three samples were stored in a well-closed, light-resistant glass vial with a 20 mL capacity. Dissolution testing was carried out on 30-day samples stored at ambient as well as refrigerated conditions (*n* = 3).

## Results and Discussion

### Screening for Printability

Various materials are suitable for additive manufacturing processes, however, the choice of the polymer used for printing is determined based on the inherent material properties and the desired characteristics of the printed object. Polymers are ideal for 3D-printed pharmaceuticals because of their vast variety and highly customizable properties. With many polymers available, it is possible to tailor formulations by adjusting the ratios of polymer mixtures to control drug release, optimize mechanical strength, or achieve other desired characteristics for specific applications. This flexibility enables the creation of precise, application-specific solutions in pharmaceutical 3D printing [30, 31]. Materials used for the fabrication of efficacious gummies using 3D printing need to be viscoelastic, should hydrate well, and show biocompatibility to form a firm gel [25]. Based on the required properties, polymers such as gelatin, pectin, carrageenan, xanthan gum, and HPMC were screened to formulate the 3D-printed gummy. Xylitol was used as a sweetening agent. BHA-BHT was used as a preservative. Gelatin and pectin, the two gelling agents, were combined with viscosity modifiers—carrageenan, xanthan gum, and HPMC K15M—in various combinations and assessed for their printability. Placebos were designed and tested for extrusion and printability at three different temperatures, 30°C, 35°C, and 45°C for 10 min, and at three different pressures i.e. 200, 250, and 300 kPa. Placebo formulations P1-P4 contained varying concentrations of gelatin with carrageenan. P1 and P2 failed to form a 3D structure at all the temperatures and pressures whereas P3 and P4 showed a degree of cylindrical shape retention and hence were selected for further evaluation.

Placebo formulations P5 to P8 containing pectin as a gelling agent and carrageenan as a viscosity-modifying agent failed to extrude as a semisolid, and hence were eliminated from further consideration. This could be due to the highly viscous printing inks which might cause inconsistent filling of the printing cartridge and thereby irregular flow of the pectin-based printing inks [32].

Placebos formulations P9 to P12 were designed such that the effect of the concentration of gelatin in water on HPMC could be determined on the printability. The gelling properties of HPMC K15M make it a suitable candidate to be used for gummy formulation [17]. The extrudates exhibited a flexible, thread-like structure, but this composition couldn’t maintain a cylindrical form. Consequently, formulations P9 through P12 were not selected for drug loading. This might be due to the poor cohesion between the subsequent layers as HPMC might have solidified before the deposition of the ensuing layers.

For placebo formulations – P13 to P16 – xanthan gum was chosen as the viscosity modifier as it is a thickening, and gelling agent used to form gummy-like structures [19]. However, it was observed that these mixtures had a thick, gel-like consistency that made them unsuitable for pouring into the 3D printing cartridge, leading to the elimination of formulations P13 to P16. Placebos formulations P17-P20 were prepared to study the effect of the aqueous phase composition on printing when combined with carrageenan, as placebos formulations P3 and P4 showed some degree of shape confirmation. P17, which contained a high amount of water was extruded along with the nozzle at the high temperature–pressure combination (45°C-300 kPa) as it was more flowable and had liquid consistency and failed to form any shape. On the contrary, placebo formulations P18 and P19 successfully formed the base layer but could not create the complete cylindrical shape.

To overcome this issue, placebos P21 and P22 were prepared using method 2 (carrageenan paste method) to ensure appropriate blending of carrageenan. P22 was successfully printed at 30°C-200 kPa and was loaded with the drugs. The successful printing can be attributed to the stirring and the consequent crosslink junction distribution and aeration. Stirring ensures the uniform dispersion of crosslinking sites within the carrageenan paste, enhancing the printed object's mechanical properties. Moreover, the aeration process during stirring helps minimize air bubbles in the mixture, leading to a smoother, more homogenous material, making it suitable for extrusion-based 3D printing [32–34].

The polymer concentration ratio and distribution of the viscosity modifying agent is critical to the printability and mechanical properties of the gummies. This insight can provide a foundational guideline for developing formulations with other drugs, as the balance of polymer concentration plays a pivotal role in achieving suitable rheological and textural properties for successful 3D printing.

### Physical Properties of 3D-Printed Gummies

The diameter and height were measured for all three active formulations. These results are shown in Table V.

**Table V:** Dimensions of 3D-Printed Gummy Formulations (± 5% of the Assigned Specification was Accepted)

Gummy Formulation	Diameter (mm)	Diameter specification (mm)	Height (mm)	Height specification (mm)
F1	17 ± 1.9	16 ± 0.8	11 ± 4.1	9 ± 0.45
F2	16 ± 0.1	15 ± 0.75	8 ± 1.4	8 ± 0.4
F3	14 ± 0.4	14 ± 0.7	7 ± 0.3	7 ± 0.45

The dimensions of F1 and F2 deviated from the intended printing dimensions and exhibited significant sample-to-sample variations. This could be attributed to the high viscosity of the formulations, which might have affected pressure distribution in the cartridge due to uneven filling. While F1 and F2 could be printed into the desired cylindrical shape, they showed differences in gummy weight. In contrast, formulation F3 repeatedly produced consistent results using the same parameters, demonstrating uniformity in dimensions and weight.

F3A-F3D didn’t form any shape and were printed as hard material. As the concentration of carrageenan in the gummy formulations increased, successful fabrication became difficult, resulting in only a solid, thick extrudate rather than a printable gummy. This behavior can be attributed to the rapid gelation rate and enhanced viscoelastic properties caused by the higher carrageenan concentration and thus a possible formation of additional network nodes and double helices formed by carrageenan [35]. The increased storage modulus and yield stress made the material too rigid and less flowable, impeding the extrusion process. Additionally, the higher thermal stability and the strengthened microstructure, driven by electrostatic interactions between gelatin and carrageenan, further contributed to the material's inability to retain its form during 3D printing, leading to a solid, non-printable output [36].

**Fig. 1:**
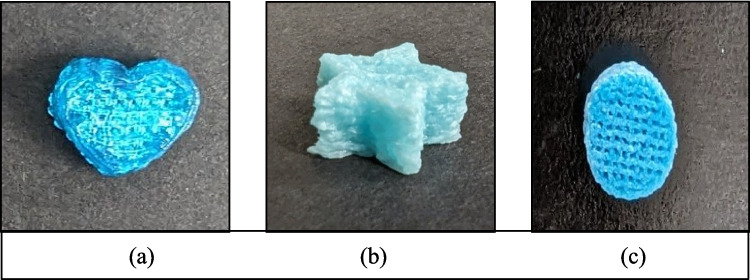
The 3D-printed gummies in different shapes (**a**) heart (**b**) star (**c**) cylinder.

The samples shown in Fig. 1 were printed to check the precision of the 3D printer and to check if the F3 gummies could be printed in different shapes such as a heart, star, and cylinder.

### Rheological Tests for Gummy Matrix

The printability of gummy-like formulations in extrusion-based 3D printing can be influenced by various factors. These factors include: 1) the initial phase of the matrix before extrusion, 2) the viscosity of the matrix, 3) rheological characteristics [37].

**Fig. 2:**
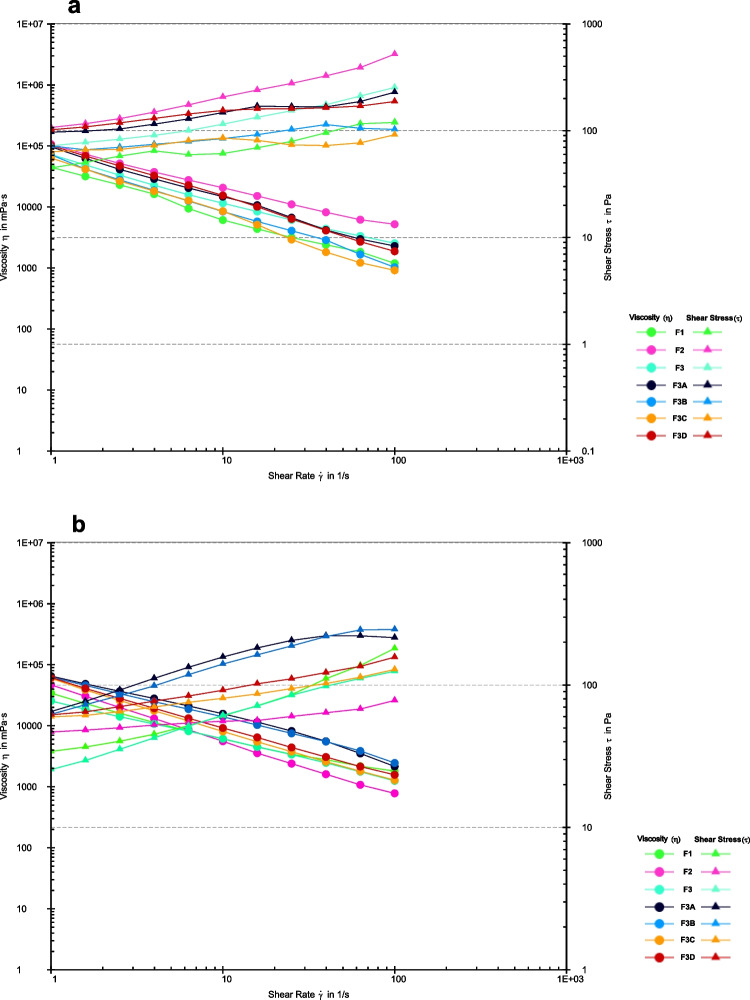
Shear stress and viscosity profiles (flow curves) of printing inks from 0.01 to 100 1/s within 120 s at a temperature of (**a**) 23°C and (**b**) 30°C.

The flow curves in Fig. 2 were measured at two different temperatures – 23°C (print bed) and 30°C (printhead). Both of the flow curves show shear thinning behavior, i.e. non-Newtonian behavior, which means the viscosity decreases as the shear strain increases [38]. This rheological property aids extrusion via a printer nozzle [25]. The viscosity of the printing inks is a critical parameter for extrusion-based additive manufacturing. When the viscosity is too low, the material tends to leak easily from the nozzle, making it challenging to achieve proper shape during printing. Conversely, if the material has a very high viscosity, it becomes semi-solid, requiring high pressure for extrusion or preventing extrusion altogether as seen for formulations F3A to F3D. The viscosity profiles for the formulations F3A-F3D show higher viscosity values and increased shear stress at the printhead temperature, thus confirming the increase in viscosity with increased carrageenan concentration which consequently causes difficulty in printing. Hence, the viscosity study performed to find suitable viscosities for different drug formulations shows that these formulations demonstrated typical shear-thinning behavior.

**Fig. 3:**
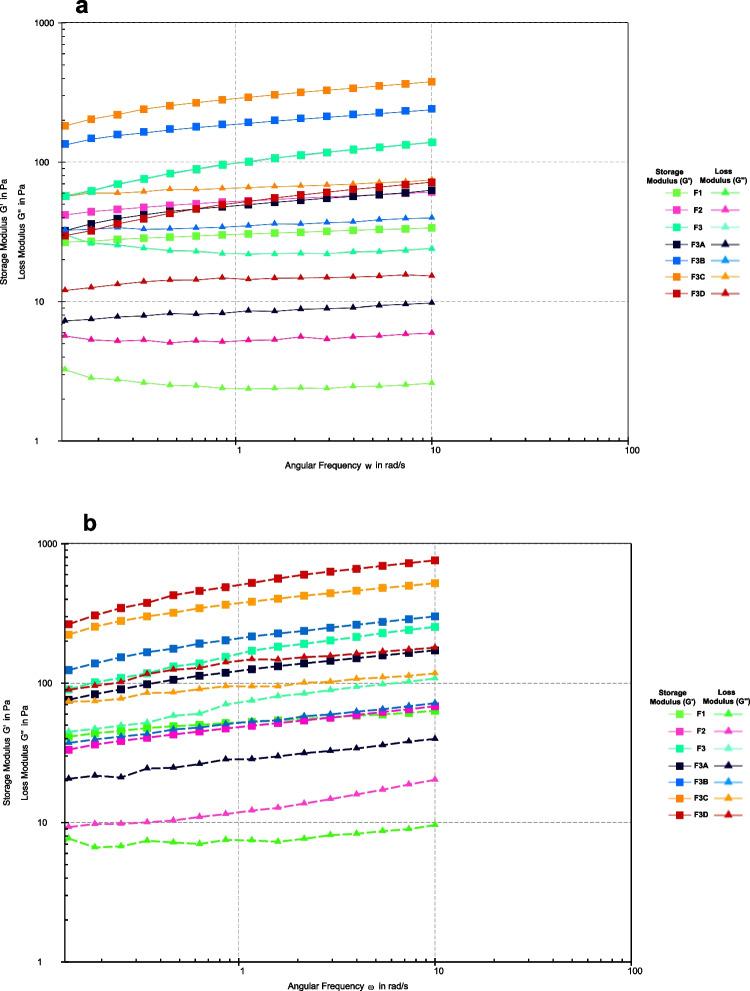
Storage modulus (G') and Loss modulus (G'') of printing inks conducted at (**a**) 23°C and (**b**) 30°C.

Figure 3 shows the frequency sweep results; all the formulations show viscoelastic properties with a gel-like behavior i.e. simultaneous viscous (liquid-like) and elastic (solid-like) properties in the formulation. The G' (storage modulus) also known as the elastic modulus values are higher than G'' (loss modulus) also known as the viscous modulus. G' shows the elastic behavior of the material when deformed and G'' reflects the flow of material while it is deformed, giving information on the behavior and inner structure of the polymers [21]. The evaluation at 30°C, the printhead temperature, reflects the material's behavior during extrusion. At this temperature, a lower G'' ensures that the material flows sufficiently through the nozzle, but the higher G' helps it transition to a solid state upon extrusion, ensuring it doesn’t spread uncontrollably.

**Fig. 4:**
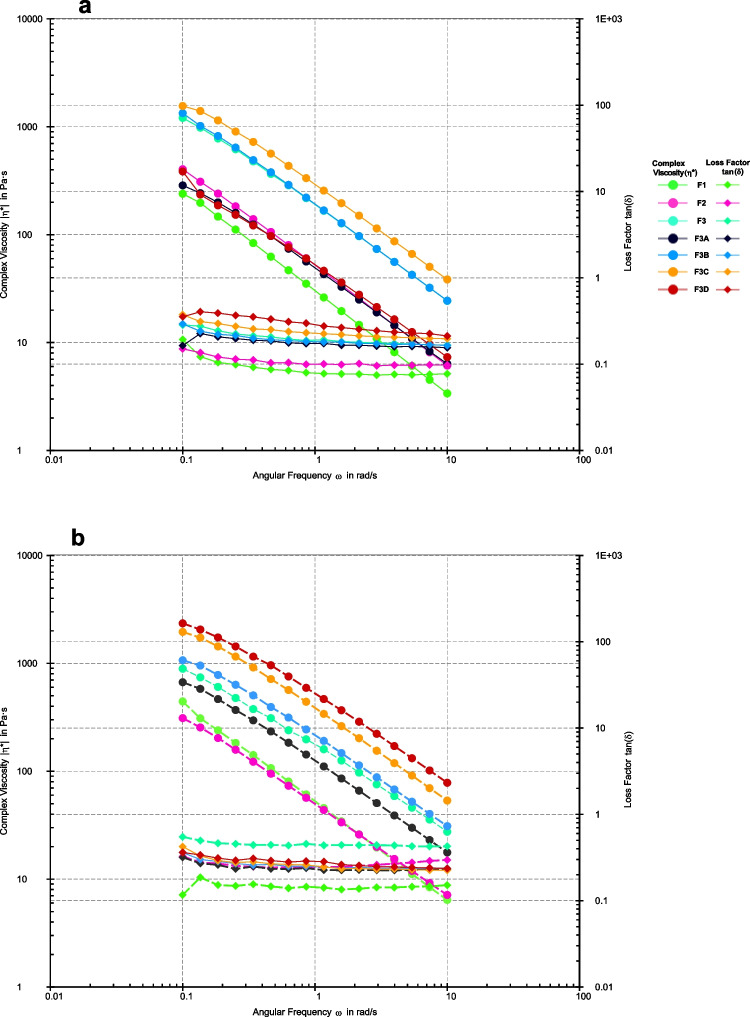
Complex viscosity and loss factor tan (δ) of different formulations conducted at (**a**) 23°C and (**b**) 30°C.

The loss factor, known as tan δ, is calculated by dividing the loss modulus (Gʺ) by the storage modulus (Gʹ) in viscoelastic materials. This ratio shows the balance between the material's viscous and elastic properties. If the tan δ value is less than 1, the material is more elastic. If the value is greater than 1, the material behaves more like a viscous fluid [39]. Hence, it can be seen in Fig. 4 that despite the temperature, the values of Tan δ are less than 1, implying that all the formulations have higher elastic character and would lead to the formation of a stiff gummy after extrusion. Moreover, decreasing complex viscosity along with increasing angular frequency demonstrates shear thinning behavior thus agreeing with the suitability of the inks to extrude under pressure.

**Fig. 5:**
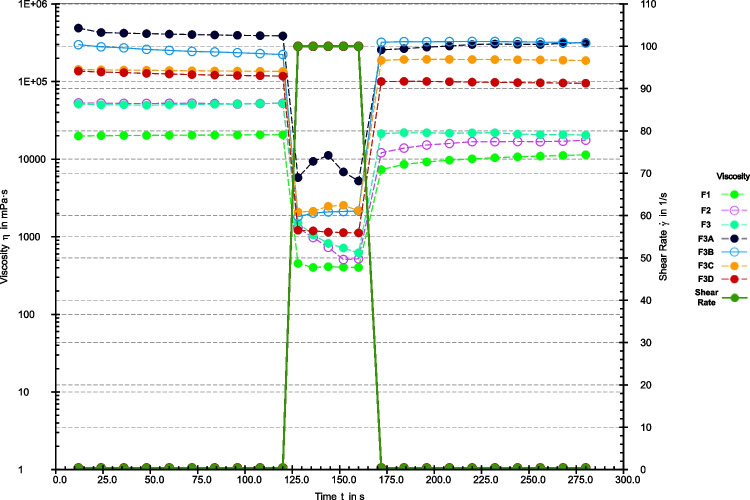
Shear recovery test for thixotropy evaluation.

Figure 5 indicates a decrease in viscosity over time when constant shear stress is applied, followed by a slow return to its original state once the stress is removed indicating the thixotropic nature of the inks [38]. Therefore, the formulations showed rapid recovery after extruding and regained shape by rapidly regaining the viscosity. All the formulations exhibited quick recovery, but formulations F3A-F3D, which were more viscous, showed a higher degree of recovery, which could corroborate the development of a solid extrudate and the failure of the formation of the gummies. In contrast, formulations F1-F3, which were less viscous after shear was applied, experienced a further decrease in viscosity, maintaining a softer, gummy-like consistency. The thixotropy recovery evaluations correspond with previous studies thereby explaining the shape fidelity of F1, F2, and F3 [40]. The increased viscosity for F3A-F3D could verify the probable development of additional network nodes that can be attributed to the gelatin-carrageenan ratios in the formulations [20].

### Fourier Transform Infrared Spectroscopy (FT-IR)

**Fig. 6:**
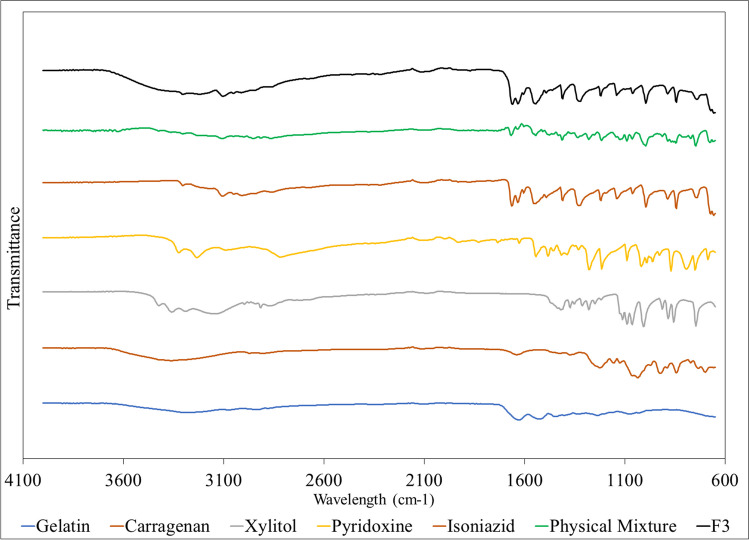
FTIR spectra of excipients, physical mixture and formulation

FTIR spectroscopy was utilized to assess the compatibility between the active pharmaceutical ingredients (APIs), and excipients used in the final 3D printed formulation (F3) (Fig. 6). The characteristic transmittance bands for each component were examined for shifts or disappearances that might indicate molecular-level interactions [26, 41]. Pure INH exhibits characteristic bands at 3303 and 3107 cm^−1^ (N–H stretching), 3010 cm^−1^ (C-H stretching), 1663 cm^−1^ (C = O stretching), and 1633 cm^−1^ (C = C stretching) [42]. PDX showed characteristic bands at 1017, 1274 and 1541 cm^−1^ originating from stretching vibrations of the pyridine ring [43]. Gelatin revealed its strong amide A band due to N–H stretching above 3000 cm^−1^ and amide I band near 1650 cm^−1^ resulting from C = O stretching. The amide II band, due to N–H bending coupled with C-N stretching, is observed around 1550 cm^−1^ [44]. FTIR spectrum of carrageenan showed characteristic bands at 3382, 1637, 1374, 1223 and 1125 cm^−1^ [45]. Furthermore, the characterization of xylitol has shown characteristic bands at 3354, 3284 cm^−1^ (O–H stretching), 1418 cm^−1^ (C-H stretching) [46]. The physical mixture and the final formulation (F3) showed the same characteristic bands at 3107, 1663, 1633, 1541, 1374, 1223, and 1125 cm^−1^. In the case of the final formulation (F3), the results indicated that INH and PDX did not interact with the excipients under the conditions applied in the formulation process, affirming the suitability of the 3D printing process for this formulation and the stability of the APIs within the matrix.

### Drug Content

The drug content was determined for the 3D-printed gummy formulations to evaluate the amount of each drug present in the formulation. Formulation F3 had drug content within the acceptable limits according to USP guidelines, as shown in Table VI. However, since F1 and F2 had issues with weight variation that didn’t meet the requirements, the drug content for these two formulations wasn't measured.

**Table VI:** Drug content of 3D-Printed Gummy Formulations (± 5% of the Assigned Theoretical Weight was Accepted)

Formulation	Theoretical Weight (gm)	Practical Weight (gm)	Drug Content (%)
F1-INH	3.6 ± 0.18	1.71 ± 2.45	-
F1- PDX			-
F2-INH	1.8 ± 0.09	1.24 ± 2.01	-
F2-PDX			-
F3-INH	1.2 ± 0.06	1.19 ± 1.25	99 ± 5%
F3-PDX			99 ± 5%

### Texture Profile Analysis

Texture properties are important attributes of the gummies as they impact the patient acceptance in terms of the mouthfeel and ease of chewing. Hardness reflects the gummy’s ability to resist deformation under force, much like the resistance experienced when chewing, and its value is estimated from the maximum force on the first compression. While a firm structure is necessary to prevent breaking during handling, a softer texture is crucial for ensuring patient compliance, making it more suitable for all age groups [40]. In this study (Table VII), the hardness value of the printed gummy (F3) displayed a slightly lower hardness value of 382.6 ± 42.8 g compared to the comparator hardness of 467.2 ± 15.4 (P = 0.048). Springiness is a measure of a gummy's elasticity or its ability to return to its original height after the first compression which mirrors the rubbery sensation experienced in the mouth during chewing. Higher springiness values indicate the gummy breaks into larger pieces after the initial bite, conversely, lower springiness values indicate breaking into smaller pieces [20]. F3's springiness value was 1.95 ± 0.16 mm, comparable to the comparator's 1.79 ± 0.28 mm (P > 0.05), indicating comparable elasticity and a similar mouthfeel between F3 and the comparator. Cohesiveness refers to how well the gummy particles hold together under compression, calculated from the ratio of work done during the second compression cycle to the first. F3 exhibited a cohesiveness value of 0.68 ± 0.04, which was lower than the comparator value of 0.95 ± 0.005 (P > 0.05). However, this lower cohesiveness is desirable, as it allows for easier disintegration of the gummy during chewing, improving both chewability and the overall consumer experience. Gumminess, the product of hardness and cohesiveness, was measured at 257.9 ± 13.7 for F3, lower than the comparator's 442.3 ± 16.9. Similarly, chewiness, a combination of hardness, cohesiveness, and springiness, was 502.7 ± 14.7 for F3, compared to 652.1 ± 278.9 for the comparator. The texture parameters would classify the printed gummies as moderately firm and chewy compared to the comparator which was firm and very chewy based on the tactility scale by Santamaria *et al*., [47]. Overall, F3 demonstrates a unique combination of textural properties including moderate hardness, suitable springiness, and desirable cohesiveness for disintegration, all of which make it an ideal formulation for consumer satisfaction and patient compliance.

**Table VII:** Texture Properties of the Formulation and the Comparator

Texture Properties	F3	Comparator
Hardness (g)	382.61 ± 42.77	467.19 ± 15.41
Springiness (mm)	1.95 ± 0.16	1.79 ± 0.28
Cohesiveness	0.68 ± 0.03	0.947 ± 0.005
Gumminess	257.9 ± 13.65	442.29 ± 16.86
Chewiness	502.72 ± 14.68	918.42 ± 86.43

## *In Vitro* Drug Release

**Fig. 7:**
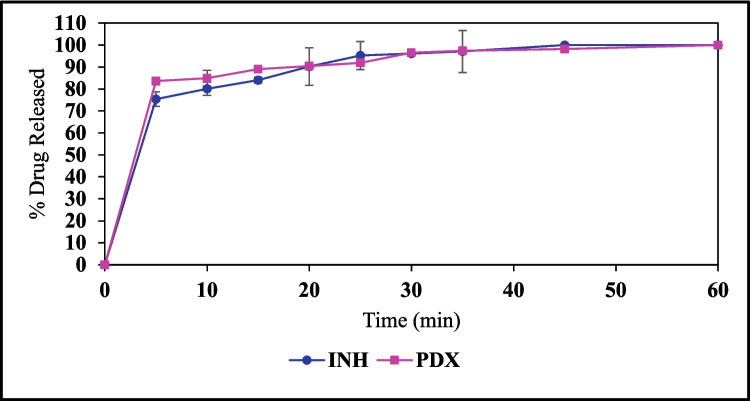
Dissolution profile of 3D-printed gummy formulation

The INH and PDX release profile of formulation F3 was investigated under pH 1.2 to mimic the physiological condition of the stomach. The release of the drugs was measured at 5, 10, 15, 20, 25, 30, 35, 45 and 60-min intervals (Fig. 7). INH initially exhibited a rapid release, reaching an average of 75.39% ± 3.29 by the first 5 min and attaining a 100% release by 45 min. Meanwhile, PDX showed a slightly quicker release, starting at 83.62% ± 1.42 at 5 min, and culminating in full release by the 60-min mark. The immediate release can be attributed to the hydrophilic matrix of the formulation made up of water-soluble excipients. The results are consistent with prior studies performed with gummies comprising gelatin matrices [17, 18].

### Stability Studies

**Fig. 8:**
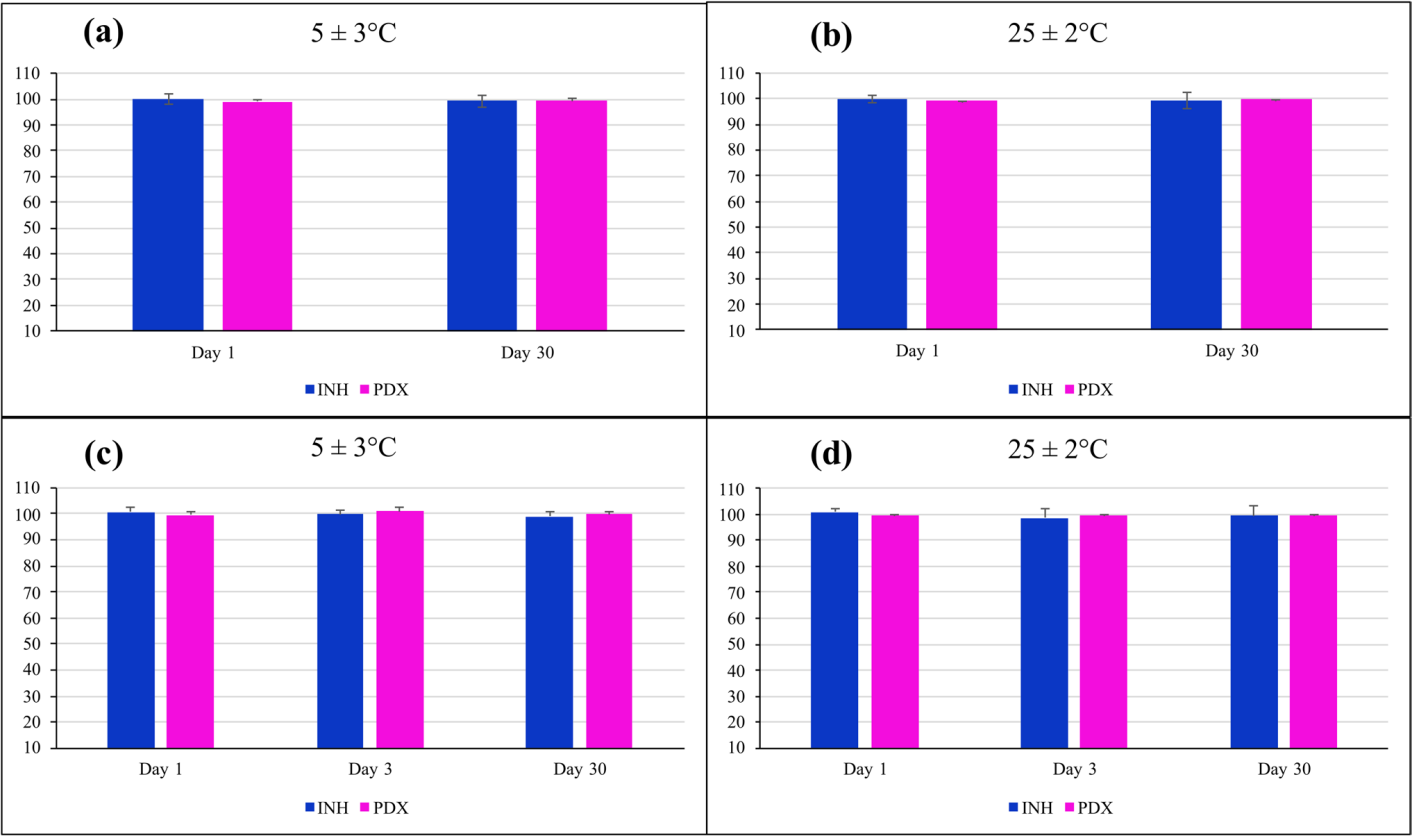
Drug content of the 3D printed gummies at (**a**) 5 ± 3°C and (**b**) 25 ± 2°C; and the gummy matrix stored in printing cartridges at (**c**) 5 ± 3°C and (**d**) 25 ± 3°C for 30 days.

To assess the stability of the 3D-printed INH-PDX gummy formulation F3 and its gummy matrix under different environmental conditions, the drug content and release profiles were monitored at regular intervals for 30 days. The drug content stayed over 95% in all conditions, again demonstrating the stability of both the drugs in the formulation over the 30 days (Fig. 8a and b). The stability of the gummy matrix stored in the printing cartridge was also studied for drug content over Day 1, Day 3, and Day 30 (Fig. 8c and d). The drug content for the gummy matrix remained above 95% in all conditions, again demonstrating minimal degradation of INH and PDX in the formulation over the 30 days. These findings show that the gummy matrix can be stored in a printing cartridge for 30 days for on-demand printing. However additional studies need to be conducted on studying microbial growth in the matrix and if any incompatible interactions occur when the material is stored in the cartridge.

**Fig. 9:**
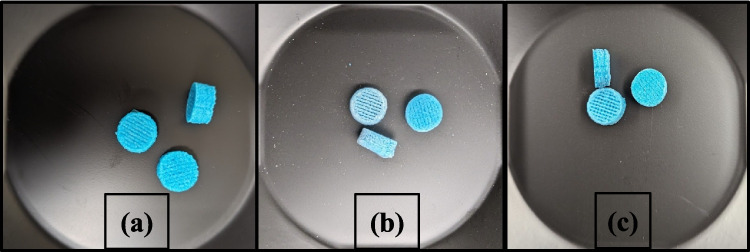
3D-printed isoniazid-pyridoxine gummies at (**a**) day 1; (**b**) day 30 – 25 ± 2°C; and (**c**) day 30 – 5 ± 3°C.

The formulation showed good integrity and shape fidelity even after 30 days. This indicates the formulations contained an optimum amount of water and the gummies did not shrink in size [38]. However, a slight variation was seen in the physical appearance of the formulations. The formulations stored at 25 ± 2°C showed a white residue (Fig. 9b). This white residue can be attributed to the “blooming” phenomenon observed with plasticizers such as xylitol and sorbitol [48, 49]. The plasticizer is thrown out of the formulation due to solubility issues from storage in ambient conditions. This finding is consistent with the other studies where white residues have been observed upon storage [50, 51]. However, the formulations stored at 5 ± 3°C had no residue and remained visually appealing. These findings suggest that the suitable storage condition for this formulation would be 5 ± 3°C.

**Fig. 10:**
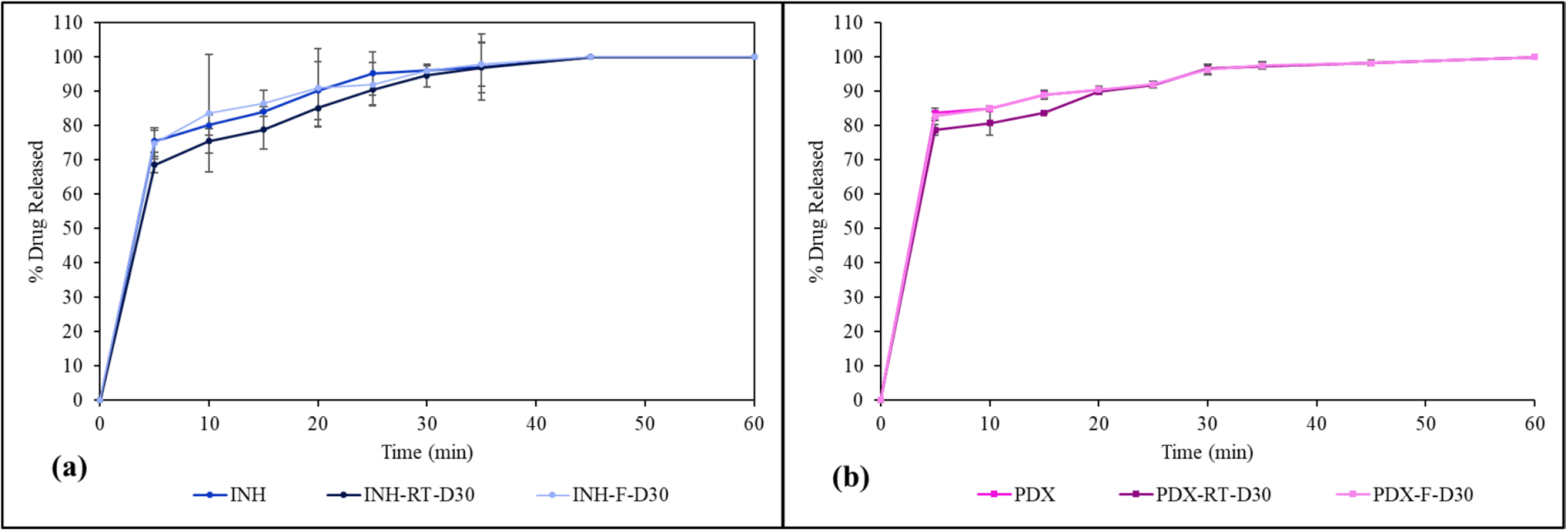
Dissolution profile of (**a**) isoniazid and (**b**) pyridoxine in 3D-printed gummy formulation F3 at 25°C ± 2°C (RT) and 5°C ± 3°C (F).

The release of INH and PDX from the 3D-printed gummies was investigated under two storage conditions: room temperature (RT) 25 ± 2°C and refrigerated temperature (F) 5 ± 3°C; 30 days post-printing and the percentage drug release for both the drugs was compared against the *in vitro* release calculated at day 1 (Fig. 10). As described in the section above, both the drugs showed more than 80% release within 30 min on day 1, with INH showing a 96.085 ± 1.02% release, while 96.49 ± 1.49% PDX was released from the formulation.

For the gummies stored in refrigerated conditions over 30 days, the release rate of both the drugs remained almost similar to day 1 drug release, with 96.08 ± 1.29% and 96.2 ± 1.47% for INH and PDX respectively. At 45 min, INH was completely released from the F3 gummies stored at refrigerated conditions. PDX was completely released in 60 min. The gummies stored at refrigerated conditions over 30 days thus showed a similar release pattern to the gummies evaluated on day 1. At room temperature, INH's release at 5 min was slightly lower on day 30 than on day 1, with an average of 68.58 ± 2.40%, suggesting a slight decrease in the release rate over time. PDX also showed a reduced drug release (77.65 ± 1.58%) at the 5-min mark. The room temperature condition showed a slight retardation effect for the initial time points, however, the release caught up to the day 0 release at the 30 min mark. INH and PDX both released 94.6 ± 3.29% and 96.49 ± 1.45% respectively at 30 min from the gummies stored at ambient conditions, thus displaying an immediate release behavior.

## Conclusion

Dual-drug loaded immediate-release 3D-printed gummy formulations were successfully developed using Semi-Solid Extrusion with the Bio X 3D Printer. FT-IR analysis confirmed that there were no incompatibilities between the drugs or with the excipients. Rheological testing revealed that the premix exhibited shear thinning, viscoelastic, and thixotropic properties, which are crucial for maintaining the gummy’s structure. Texture analysis showed that F3 offers an ideal blend of texture with moderate hardness, suitable springiness, and cohesive disintegration. The gelatin-to-carrageenan ratio in formulation F3 was found to be the most suitable for producing gummies via 3D printing. Stability tests showed that F3 remained stable for 30 days at both 25°C ± 2°C and 5°C ± 3°C, although storing it at 5°C ± 3°C is advised to preserve its visual appeal. The premix can also be stored in the cartridge for 30 days and printed as needed. The 3D-printed formulations can be customized into any shape, making them an appealing dosage form. Future studies are needed to investigate the residue observed and to determine if the cartridge material is appropriate for long-term storage of the premix.

## Data Availability

All data generated or analyzed during the study are included in this article.

## References

[1] Tobin EH, Tristram D. Tuberculosis. In: StatPearls [Internet]. Treasure Island (FL): StatPearls Publishing; 2024. http://www.ncbi.nlm.nih.gov/books/NBK441916/.

[2] Centers for Disease Control and Prevention. About Tuberculosis [Internet]. Tuberculosis (TB). 2024. https://www.cdc.gov/tb/about/index.html. Accessed 22 Jul 2024.

[3] Harries AD, Kumar AMV, Satyanarayana S, Thekkur P, Lin Y, Dlodlo RA, *et al*. The Growing Importance of Tuberculosis Preventive Therapy and How Research and Innovation Can Enhance Its Implementation on the Ground. Trop Med Infect Dis. 2020;5(2):61. 10.3390/tropicalmed5020061. 32316300 10.3390/tropicalmed5020061 PMC7345898

[4] World Health Organization. 3. TB prevention and screening [Internet]. 2024. https://www.who.int/teams/global-tuberculosis-programme/tb-reports/global-tuberculosis-report-2023/tb-prevention.

[5] Tayal A, Kabra SK. Tuberculosis Preventive Treatment. Indian J Pediatr. 2024;91:823–9. 10.1007/s12098-023-04969-z. 38095783 10.1007/s12098-023-04969-z

[6] Goyanes A, Madla CM, Umerji A, Duran Piñeiro G, Giraldez Montero JM, Lamas Diaz MJ, *et al*. Automated therapy preparation of isoleucine formulations using 3D printing for the treatment of MSUD: First single-centre, prospective, crossover study in patients. Int J Pharm. 2019;567: 118497. 10.1016/j.ijpharm.2019.118497. 31279771 10.1016/j.ijpharm.2019.118497

[7] Vaz VM, Kumar L. 3D Printing as a Promising Tool in Personalized Medicine. AAPS PharmSciTech. 2021;22(1):49. 10.1208/s12249-020-01905-8. 33458797 10.1208/s12249-020-01905-8 PMC7811988

[8] Huanbutta K, Burapapadh K, Sriamornsak P, Sangnim T. Practical Application of 3D Printing for Pharmaceuticals in Hospitals and Pharmacies. Pharm. 2023;15(7):1877. 10.3390/pharmaceutics15071877. 10.3390/pharmaceutics15071877 PMC10385973 37514063

[9] Fastø MM, Genina N, Kaae S, Kälvemark SS. Perceptions, preferences and acceptability of patient designed 3D printed medicine by polypharmacy patients: a pilot study. Int J Clin Pharm. 2019;41(5):1290–8. 10.1007/s11096-019-00892-6. 31444687 10.1007/s11096-019-00892-6

[10] Han X, Kang D, Liu B, Zhang H, Wang Z, Gao X, *et al*. Feasibility of developing hospital preparation by semisolid extrusion 3D printing: personalized amlodipine besylate chewable tablets. Pharm Dev Technol. 2022;27(2):164–74. 10.1080/10837450.2022.2027965. 35007187 10.1080/10837450.2022.2027965

[11] Seoane-Viaño I, Januskaite P, Alvarez-Lorenzo C, Basit AW, Goyanes A. Semi-solid extrusion 3D printing in drug delivery and biomedicine: Personalised solutions for healthcare challenges. J Control Release. 2021;10(332):367–89. 10.1016/j.jconrel.2021.02.027. 10.1016/j.jconrel.2021.02.027 33652114

[12] Mafukidze AT, Calnan M, Furin J. Peripheral neuropathy in persons with tuberculosis. J Clin Tuberc Mycobact Dis. 2016;2:5–11. 10.1016/j.jctube.2015.11.002. 10.1016/j.jctube.2015.11.002 PMC6852705 31768422

[13] Snider DE. Pyridoxine supplementation during isoniazid therapy. Tubercle. 1980;61(4):191–6. 10.1016/0041-3879(80)90038-0. 6269259 10.1016/0041-3879(80)90038-0

[14] Badrinath M, Chen P, John S. Isoniazid Toxicity. In: StatPearls [Internet]. Treasure Island (FL): StatPearls Publishing; 2024. http://www.ncbi.nlm.nih.gov/books/NBK531488/. 30285383

[15] van Galen KA, Nellen JF, Nieuwkerk PT. The Effect on Treatment Adherence of Administering Drugs as Fixed-Dose Combinations versus as Separate Pills: Systematic Review and Meta-Analysis. AIDS Res Treat. 2014. 10.1155/2014/967073. 25276422 10.1155/2014/967073 PMC4168145

[16] Ting NCH, El-Turk N, Chou MSH, Dobler CC. Patient-perceived treatment burden of tuberculosis treatment. PLoS ONE. 2020;15(10): e0241124. 10.1371/journal.pone.0241124. 33091084 10.1371/journal.pone.0241124 PMC7580887

[17] Tagami T, Ito E, Kida R, Hirose K, Noda T, Ozeki T. 3D printing of gummy drug formulations composed of gelatin and an HPMC-based hydrogel for pediatric use. Int J Pharm. 2021;1(594): 120118. 10.1016/j.ijpharm.2020.120118. 10.1016/j.ijpharm.2020.120118 33326827

[18] Zhu C, Tian Y, Zhang E, Gao X, Zhang H, Liu N, *et al*. Semisolid Extrusion 3D Printing of Propranolol Hydrochloride Gummy Chewable Tablets: an Innovative Approach to Prepare Personalized Medicine for Pediatrics. AAPS PharmSciTech. 2022;23(5):166. 10.1208/s12249-022-02304-x. 35705726 10.1208/s12249-022-02304-x

[19] García-Segovia P, García-Alcaraz V, Balasch-Parisi S, Martínez-Monzó J. 3D printing of gels based on xanthan/konjac gums. Innov Food Sci Emerg Technol. 2020;1(64): 102343. 10.1016/j.ifset.2020.102343.

[20] Kuo CC, Qin H, Cheng Y, Jiang X, Shi X. An integrated manufacturing strategy to fabricate delivery system using gelatin/alginate hybrid hydrogels: 3D printing and freeze-drying. Food Hydrocoll. 2021;1(111): 106262. 10.1016/j.foodhyd.2020.106262.

[21] Xie F. Chapter 4 - 3D printing of biopolymer-based hydrogels. In: Mehrpouya M, Vahabi H, editors. Additive Manufacturing of Biopolymers. Elsevier; 2023. pp. 65–100. 10.1016/B978-0-323-95151-7.00004-1.

[22] Wang H, Karnik I, Uttreja P, Zhang P, Vemula SK, Repka MA. Development of Mathematical Function Control-Based 3D Printed Tablets and Effect on Drug Release. Pharm Res. 2024. 10.1007/s11095-024-03780-5. 39433693 10.1007/s11095-024-03780-5 PMC11599347

[23] Bhatkande A, Narala S, Wang H, Narala N, Karnik I, Vemula SK, *et al*. Extrusion-Based Three-Dimensional Printing of Metronidazole Immediate Release Tablets: Impact of Processing Parameters and in Vitro Evaluation. J Pharm Innov. 2024;19(6):72. 10.1007/s12247-024-09878-y.

[24] Januskaite P, Xu X, Ranmal SR, Gaisford S, Basit AW, Tuleu C, *et al*. I Spy with My Little Eye: A Paediatric Visual Preferences Survey of 3D Printed Tablets. Pharm. 2020;12(11):1100. 10.3390/pharmaceutics12111100. 10.3390/pharmaceutics12111100 PMC7698452 33212847

[25] Herrada-Manchón H, Rodríguez-González D, Alejandro Fernández M, Suñé-Pou M, Pérez-Lozano P, García-Montoya E, *et al*. 3D printed gummies: Personalized drug dosage in a safe and appealing way. Int J Pharm. 2020;25(587): 119687. 10.1016/j.ijpharm.2020.119687. 10.1016/j.ijpharm.2020.119687 32730802

[26] Uttreja P, Youssef AAA, Karnik I, Sanil K, Narala N, Wang H, *et al*. Formulation Development of Solid Self-Nanoemulsifying Drug Delivery Systems of Quetiapine Fumarate via Hot-Melt Extrusion Technology: Optimization Using Central Composite Design. Pharm. 2024;16(3):324. 10.3390/pharmaceutics16030324. 10.3390/pharmaceutics16030324 PMC10974971 38543219

[27] Isoniazid Tablets, United States Pharmacopeia, Rockville, MD. 2024. 10.31003/USPNF_M43010_02_01. Accessed 1 Aug 2024.

[28] Pyridoxine Hydrochloride Tablets, United States Pharmacopeia, Rockville, MD. 2024. 10.31003/USPNF_M72070_01_01. Accessed 1 Aug 2024.

[29] 〈2040〉 Disintegration and Dissolution of Dietary Supplements, United States Pharmacopeia, Rockville, MD. 2024. 10.31003/USPNF_M99970_04_01. Accessed 3 Aug 2024.

[30] Ortiz-Acosta D, Moore T. Functional 3D Printed Polymeric Materials. In: Functional Materials. IntechOpen; 2019. 10.5772/intechopen.80686.

[31] Azad MA, Olawuni D, Kimbell G, Badruddoza AZM, Hossain MdS, Sultana T. Polymers for Extrusion-Based 3D Printing of Pharmaceuticals: A Holistic Materials–Process Perspective. Pharm. 2020;12(2):124. 10.3390/pharmaceutics12020124. 10.3390/pharmaceutics12020124 PMC7076526 32028732

[32] Vancauwenberghe V, Katalagarianakis L, Wang Z, Meerts M, Hertog M, Verboven P, *et al*. Pectin based food-ink formulations for 3-D printing of customizable porous food simulants. Innov Food Sci Emerg Technol. 2017;1(42):138–50. 10.1016/j.ifset.2017.06.011.

[33] Leng DE, Calabrese RV. Immiscible Liquid–Liquid Systems. In: Handbook of Industrial Mixing . John Wiley & Sons, Ltd; 2003. pp. 639–753. 10.1002/0471451452.ch12.

[34] Chesterton AKS, de Abreu DAP, Moggridge GD, Sadd PA, Wilson DI. Evolution of cake batter bubble structure and rheology during planetary mixing. Food Bioprod Process. 2013;91(3):192–206. 10.1016/j.fbp.2012.09.005.

[35] Derkach SR, Voron’ko NG, Maklakova AA, Kondratyuk YuV. The rheological properties of gelatin gels containing κ-carrageenan. The role of polysaccharide. Colloid J. 2014;76(2):146–52. 10.1134/S1061933X14020021.

[36] Derkach SR, Ilyin SO, Maklakova AA, Kulichikhin VG, Malkin AY. The rheology of gelatin hydrogels modified by κ-carrageenan. LWT - Food Sci Technol. 2015;63(1):612–9. 10.1016/j.lwt.2015.03.024.

[37] Liu Z, Zhang M, Bhandari B, Wang Y. 3D printing: Printing precision and application in food sector. Trends Food Sci Technol. 2017;1(69):83–94. 10.1016/j.tifs.2017.08.018.

[38] Zhang B, Belton P, Yi Teoh X, Gleadall A, Bibb R, Qi S. An investigation into the effects of ink formulations of semi-solid extrusion 3D printing on the performance of printed solid dosage forms. J Mater Chem B. 2024;12(1):131–44. 10.1039/D3TB01868G. 10.1039/d3tb01868g 38050731

[39] Picout DR, Ross-Murphy SB. Rheology of Biopolymer Solutions and Gels. Sci World J. 2003;24(3):105–21. 10.1100/tsw.2003.15. 10.1100/tsw.2003.15 PMC5974903 12806124

[40] Ganatra P, Jyothish L, Mahankal V, Sawant T, Dandekar P, Jain R. Drug-loaded vegan gummies for personalized dosing of simethicone: A feasibility study of semi-solid extrusion-based 3D printing of pectin-based low-calorie drug gummies. Int J Pharm. 2024;15(651): 123777. 10.1016/j.ijpharm.2024.123777. 10.1016/j.ijpharm.2024.123777 38181992

[41] Karnik I, Youssef AAA, Joshi P, Munnangi SR, Narala S, Varner C, *et al*. Formulation development and characterization of dual drug loaded hot-melt extruded inserts for better ocular therapeutic outcomes: Sulfacetamide/prednisolone. J Drug Deliv Sci Technol. 2023;12: 104558. 10.1016/j.jddst.2023.104558.

[42] Jaganathan L, Meenakshi R, Gunasekaran S, Srinivasan S. FTIR, FT-Raman spectra and quantum chemical studies of nebivolol. Mol Simul. 2011;37(12):1044–52. 10.1080/08927022.2011.589049.

[43] Radoman TS, Džunuzović JV, Grgur BN, Gvozdenović MM, Jugović BZ, Miličević DS, *et al*. Improvement of the epoxy coating properties by incorporation of polyaniline surface treated TiO2 nanoparticles previously modified with vitamin B6. Prog Org Coat. 2016;1(99):346–55. 10.1016/j.porgcoat.2016.06.014.

[44] Kemençe N, Bölgen N. Gelatin- and hydroxyapatite-based cryogels for bone tissue engineering: synthesis, characterization, in vitro and in vivo biocompatibility. J Tissue Eng Regen Med. 2017;11(1):20–33. 10.1002/term.1813. 23997022 10.1002/term.1813

[45] Arockia Mary I, Selvanayagam S, Selvasekarapandian S, Srikumar SR, Ponraj T, Moniha V. Lithium ion conducting membrane based on K-carrageenan complexed with lithium bromide and its electrochemical applications. Ionics. 2019;25(12):5839–55. 10.1007/s11581-019-03150-x.

[46] Matawo N, Adeleke OA, Wesley-Smith J. Optimal Design, Characterization and Preliminary Safety Evaluation of an Edible Orodispersible Formulation for Pediatric Tuberculosis Pharmacotherapy. Int J Mol Sci. 2020;21(16):5714. 10.3390/ijms21165714. 32784947 10.3390/ijms21165714 PMC7460872

[47] Santamaría KJ, Anaya BJ, Lalatsa A, González-Barranco P, Cantú-Cárdenas L, Serrano DR. Engineering 3D Printed Gummies Loaded with Metformin for Paediatric Use. Gels. 2024;10(10):620. 10.3390/gels10100620. 39451273 10.3390/gels10100620 PMC11507287

[48] Aulton ME, Abdul-Razzak MH, Hogan JE. The Mechanical Properties of Hydroxypropylmethylcellulose Films Derived from Aqueous Systems Part 1: The Influence of Plasticisers. Drug Dev Ind Pharm. 1981;7(6):649–68. 10.3109/03639048109055689.

[49] Rowlands AS, Lim SA, Martin D, Cooper-White JJ. Polyurethane/poly(lactic-co-glycolic) acid composite scaffolds fabricated by thermally induced phase separation. Biomaterials. 2007;28(12):2109–21. 10.1016/j.biomaterials.2006.12.032. 17258315 10.1016/j.biomaterials.2006.12.032

[50] Talja RA, Helén H, Roos YH, Jouppila K. Effect of various polyols and polyol contents on physical and mechanical properties of potato starch-based films. Carbohydr Polym. 2007;67(3):288–95. 10.1016/j.carbpol.2006.05.019.

[51] Muscat D, Adhikari B, Adhikari R, Chaudhary DS. Comparative study of film forming behaviour of low and high amylose starches using glycerol and xylitol as plasticizers. J Food Eng. 2012;109(2):189–201. 10.1016/j.jfoodeng.2011.10.019.

